# Is surgery recommended in adults with neglected congenital muscular torticollis? A prospective study

**DOI:** 10.1186/1471-2474-9-158

**Published:** 2008-11-26

**Authors:** Farzad Omidi-Kashani, Ebrahim G Hasankhani, Reza Sharifi, Mahdi Mazlumi

**Affiliations:** 1 Department of Orthopedic Surgery, Ghaem hospital, Mashhad, Iran; 2 Department of Orthopedic Surgery, Imam Reza hospital, Mashhad, Iran; 3 Department of Orthopedic Surgery, Emdadi hospital, Mashhad, Iran

## Abstract

**Background:**

Congenital muscular torticollis is the third most common congenital musculoskeletal anomaly after dislocation of the hip and clubfoot. When diagnosed early, it is obvious that it can be managed with good or excellent results. The aim of this prospective study was to determine the efficacy of surgery in neglected adult cases.

**Methods:**

From January 2003 to June 2007, 18 adult skeletally matured patients were surgically treated for neglected congenital muscular torticollis and prospectively followed (at least one year). Bipolar release was performed in all patients. Radiography and the modified Lee's scoring system which included function and cosmesis, were used to measure the surgical results. Complications were also recorded.

**Results:**

Four cases were lost during follow-up. Of the remaining 14 patients, 10 cases were males and 4 females. The age at operation ranged from 18 to 32 (average 21.9) years. The mean follow-up period was 2.5 years (range 1–5 years). Excellent results were noted in 7 patients, good in 5, and poor in 2 patients. Significant improvement (>10°) of the cervico-thoracic scoliosis was noted only in 3 of 10 patients.

**Conclusion:**

Patients with congenital muscular torticollis can benefit from surgical treatment even in adulthood. Surgical bipolar sectioning of the sternocleidomastoid muscle should be considered even in adults with irreversible facial and skeletal deformities. The surgery restores the range of neck motion and resolves the head tilt; therefore it can improve the quality of life. This procedure is an effective and relatively complication-free method.

## Background

Torticollis in Latin means twisted neck and at first Tubby in 1912 defined it as "a deformity, congenital or acquired, characterized by lateral inclination of the head to the shoulder, with torsion of the neck and deviation of the face" [1]. The term congenital muscular torticollis (CMT), a neck deformity primarily involves shortening of the sternocleidomastoid muscle that leads the head to turn toward the affected side and the chin to point to the opposite side, is its most common form and clearly should be differentiated from many other congenital and acquired types of torticollis (Table 1) [1-4].

**Table 1:** Differential diagnoses of torticollis

**Congenital:**
Muscular
Vertebral anomalies; failure of formation, segmentation or both
Ocular
**Acquired:**
Tumoral; eosinophilic granuloma, osteoma/osteoblatoma
Traumatic; C1 fracture
Inflammatory; Juvenile rheumatoid arthritis, respiratory tract infection, cervical adenitis
Hysterical
Paroxysmal torticollis of infancy
Associated with ligamentous laxity; Down syndrome

CMT is the third most common congenital musculoskeletal anomaly after dislocation of the hip and clubfoot [3], with a reported incidence of 0.3–1.9% [4,5]. Various theories have been proposed, but the true etiology of torticollis remains uncertain. As a result of fibrotic changes of the sternocleidomastoid muscle, the unilateral contracture may subsequently result in plagiocephaly, skull and facial asymmetry [6].

When diagnosed early, CMT can be managed conservatively, seldom requiring surgery. In fact, spontaneous resolution is to be expected in most patients [7,8]. In children older than one year, corrective surgery has both cosmetic and functional benefits, the best outcomes being obtained between the ages of 1 and 4 [9]. After the age of five, the form and efficacy of treatment are controversial, and little has been published on the subject and almost attributed to the older children, not adult [10-12]. Ling CM and Low YS have stated that operative treatment is of little value after this age, and the results are even worst when the operation is done after puberty and may lead to more complications [13].

Once plagiocephaly and hemihypoplasia have occurred, they cannot be corrected after maturity because of the loss of potential for growth and remodeling [11,14]. Do you still recommend surgery in this peculiar group? The aim of this prospective study was to determine the efficacy of surgery in neglected mature cases, which unfortunately, they are not rare in some places of the world.

## Methods

From January 2003 to June 2007, 18 adult skeletally matured patients were surgically treated for neglected congenital muscular torticollis and prospectively followed-up.

**Figure 1:**
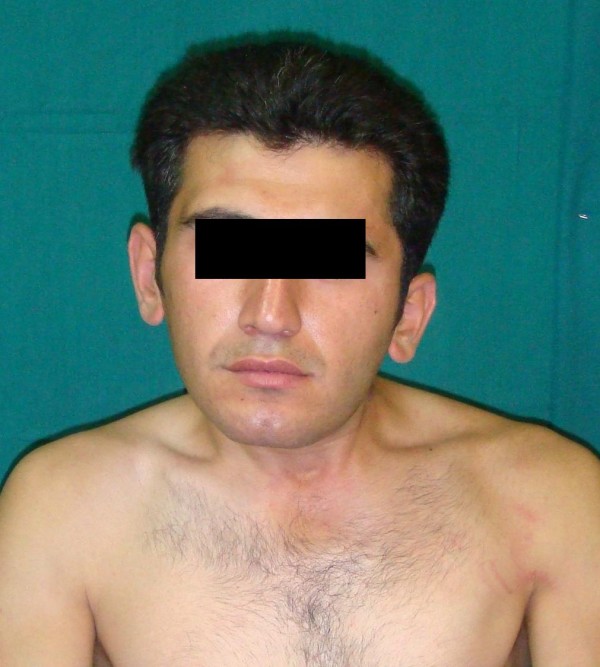
**Neglected congenital muscular torticollis**. A 25 years old man (Case 3) presented with neglected left congenital muscular torticollis.

Skeletal maturity was defined as less than 1 cm change in standing height on two consecutive measurements six months apart; Riser sign 4; or in girls, when the patient is 2 years past menarche [15]. Patients with torticollis of other etiologies and the skeletally immature cases were not included in the study. The anteroposterior and lateral radiography of the cervical spine were done in all patients, pre- and postoperatively at the last visit. The indications for surgery were a persistent head tilt (Figure 1), deficits of passive rotation and lateral bending of the neck of >15°, a tight band or tumor in the sternocleidomastoid muscle, and a poor result according to the special assessment chart [16].

The process of soft tissue healing following surgery is complex, but in normal healing, the entire process of soft tissue maturation usually takes six months to one year [17]. In regarding to the adulthood age of our patients and no expectance of craniofacial remodeling in this especial group, we considered one year as an enough period for following-up. Therefore, the cases for being included in the study must be followed at least one year after surgery.

**Figure 2:**
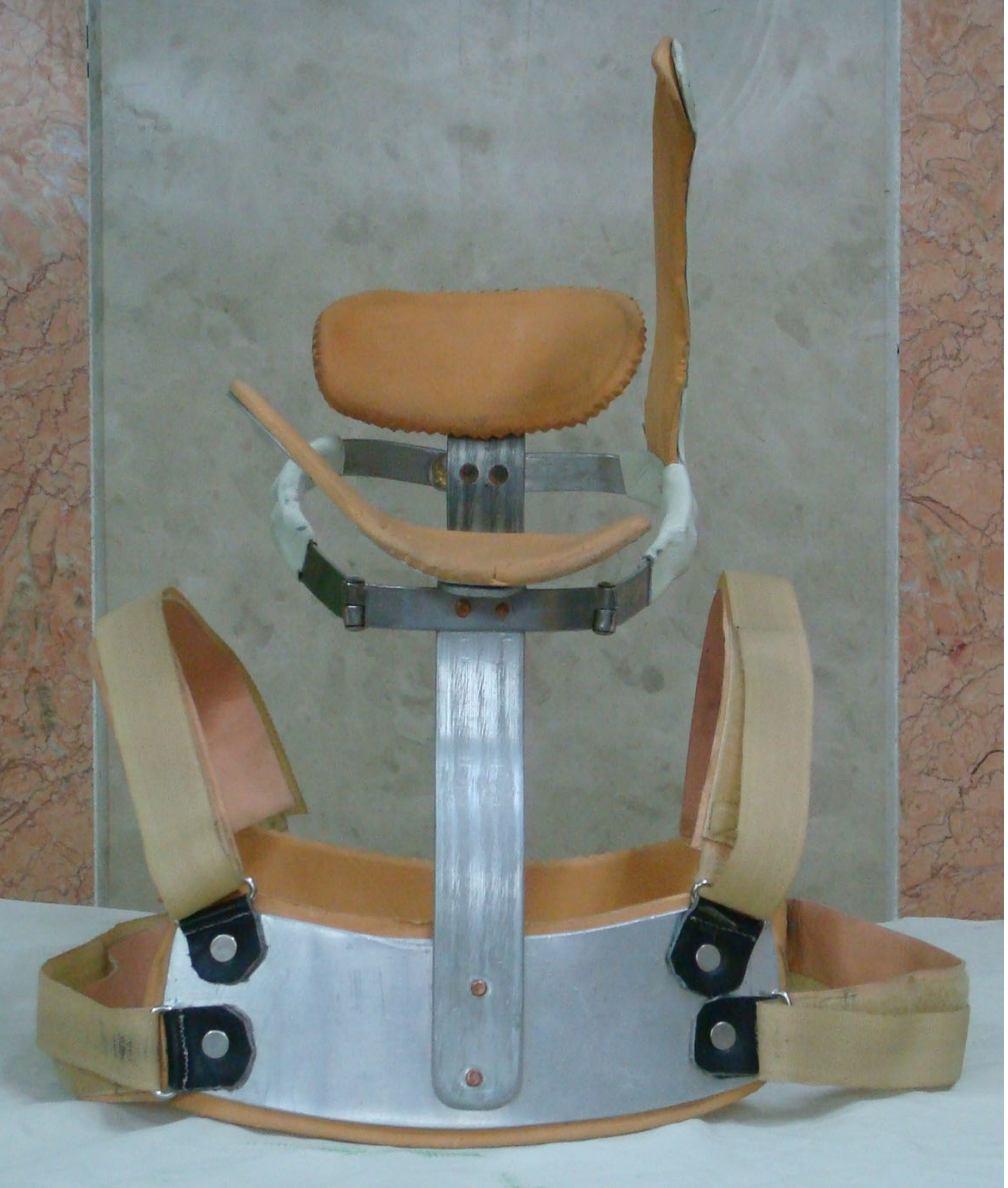
**Torticollis brace**. A form of the torticollis brace we routinely used.

**Figure 3:**
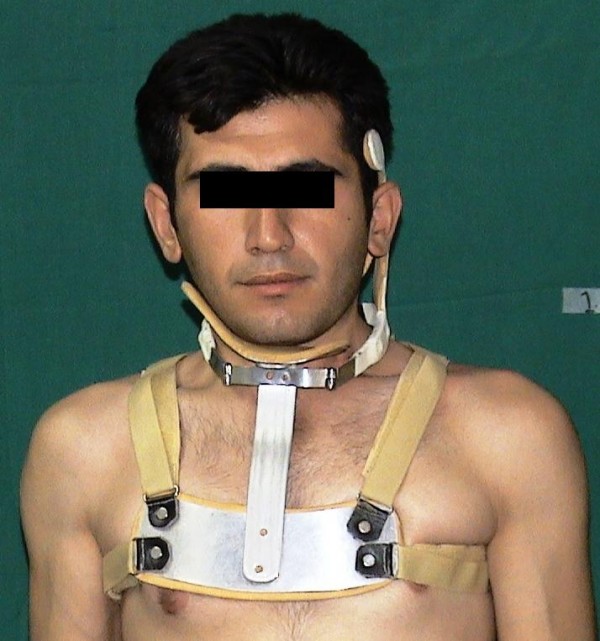
**The patient in postoperative period**. The same patient shown on figure 1, during first 3 months after surgery.

All the patients assigned the informed consents approved by local ethical committee after complete explanation about the surgery that was given by the operating surgeons themselves. A similar bipolar release technique [12] was performed in all patients by 3 different surgeons (FOK, EGH, and MM) on the same team. Postoperatively, a neck exercise program including active and passive movements was started as soon as possible and immobilization with a torticollis brace (Figure 2) was applied for at least 3 months (Figure 3). The exercises were taught to the patients and continued for the same time.

Historically, a scoring system proposed by Lee et al. which included function and cosmetic results, has been used for assessing the surgical outcome [16]. The neck movement and lateral band were compared with the uninvolved side, and the head tilt and operative scar were evaluated by clinical observation and a questionnaire. Because of the adulthood age of our patients, we ignored facial asymmetry section from the system and used "modified Lee's scoring" for this especial group of the patients (Table 2). Therefore, scorings of the surgical outcome are changed accordingly. An excellent result was given 14–15 points; a good result 12–13 points; a fair result 10–11 points and a poor result was 9 or less, or when reoperation was needed due to recurrence.

**Table 2:** Modified Lee's scoring system for the assessment of neglected muscular torticollis in adult patients

**Points**	**Function**	**Cosmesis**			
		
	**Neck movement**	**Head tilt**	**Scar**	**Loss of column**	**Lateral band**
**3**	Full	None	Fine	None	None
**2**	Limitation of rotation or side-flexion <10°	Mild	Slight	Slight	Slight
**1**	Limitation of rotation or side-flexion 10°–25°	Moderate	Moderate	Obvious but cosmetically acceptable	Obvious but cosmetically acceptable
**0**	Limitation of rotation or side-flexion >25°	Severe	Unacceptable	Unacceptable	Unacceptable

## Results

Four cases were lost during follow-up. Of the remaining 14 patients, 10 cases were males and 4 females. The age at operation ranged from 18 to 32 (average 21.9) years. The right side was involved in six patients, and left side in eight.

**Figure 4:**
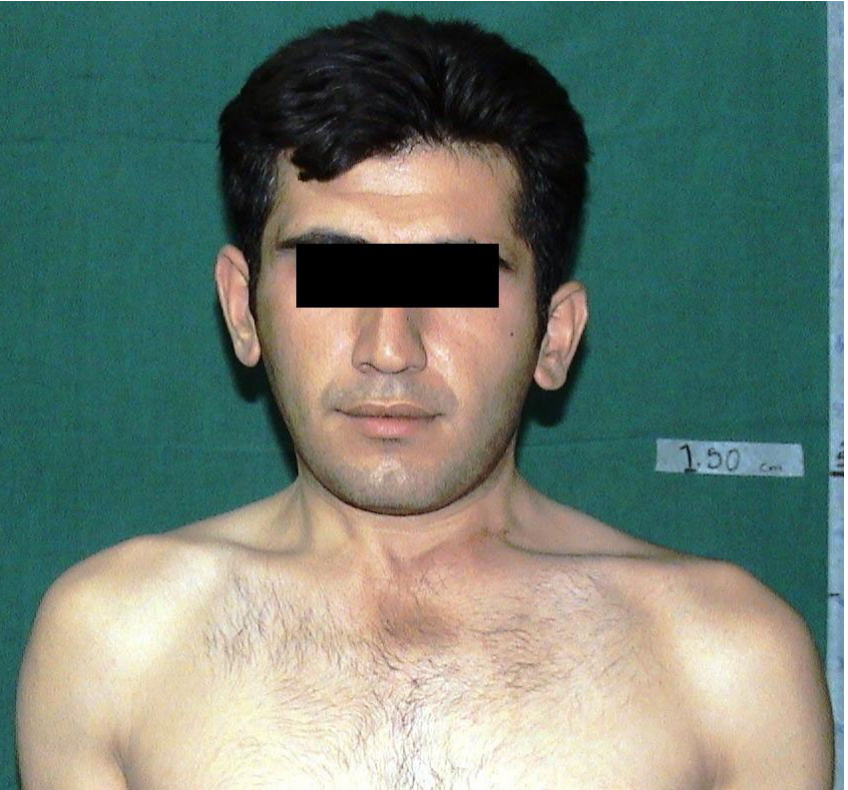
**Final photography of the patient**. The same patient shown on figure 1, 1.6 years after surgery.

The mean follow-up period was 2.5 years (range 1–5 years). According to the modified Lee's scoring system, excellent results were noted in 7, good in 5, and poor in 2 patients due to the recurrence (Table 3). No patients but two showed some improvement of the facial asymmetry (cases 2 and 11), and all of the patients except one had a less than 10° limitation of rotation of the neck movement in the latest follow-up visit (Figure 4).

**Table 3:** Summary of our patients' characteristics

Patient	Age (y)	Sex	side affected	Follow-up (y)	(Preop, postop) scoliosis	Postop points	result
1	18	F	Right	1.8	(16, 10)	13	Good
2	21	M	Right	1	(10, NS^X^)	15	Excellent
3	25	M	Left	1.6	(NS)	13	Good
4	21	M	Right	3.2	(10, NS)	14	Excellent
5	32	M	Left	2.3	(25, 12)	13	Good
6	26	M	Left	4.3	(30, 16)	8	Poor
7	27	M	Left	1.4	(NS)	15	Excellent
8	19	F	Left	3.6	(20, 10)	13	Good
9	18	F	Right	1.4	(14, NS)	15	Excellent
10	27	M	Right	5	(20, 12)	14	Excellent
11	19	M	Left	1.4	(NS)	14	Excellent
12	18	F	Left	1.8	(NS)	15	Excellent
13	25	M	Left	3.6	(26, 18)	9	Poor
14	20	M	Right	2.1	(18, 10)	13	Good

^X ^NS: Not Significant

### Complications

There were two recurrences (cases 6 and 13). Although in both of them extensive release had been carried out, we thought poor compliance of the patients with brace wearing was the main cause. Otherwise their intraoperative findings were similar to other patients we operated: One patient (case 13) was a rural bus driver. As he noted the reason for poor postoperative compliance was a variety of difficulties in driving a bus in brace. The patient refused further management. The second one (case 6) was an unmarried man with a low socioeconomic state whose main concern was cosmetic. He admitted to wear the brace regularly; therefore he was finally operated again with a similar technique. After the revision surgery, the result was good.

There was neither nerve injury (accessory or facial) nor wound infection in our series, but we had three patients with superficial hematoma, all at the proximal incision site of the operation that resolved completely with conservative measures. There was a patient with a transient tongue paresthesis that we could not realize the cause. This paresthesis disappeared on the 3^rd ^day after surgery.

The preoperative radiographs showed an average 18.9° (range, 10°–30°) of cervico-thoracic scoliosis in 10 patients. Significant improvement of the scoliotic curves (at least 10°) was noted in 3 patients. A history of congenital dislocation of the hip was noted in 2 patients (cases 8 and 12) that former was operated in childhood but the latter presented with neglected bilateral congenital hip dislocation. A history of clubfoot deformity was present in no patient.

## Discussion

Most cases of CMT resolve completely either spontaneously within months after birth or with conservative measures initiated early such as gentle controlled passive manual stretching exercises on the affected side. Sonmez K et al. found that 95% of patients diagnosed and treated effectively at the age of before one year, did not need surgical treatment [18].

In the patients seen later, surgical intervention should be considered as the treatment of choice in order to avoid further irreversible changes. Surgery is also recommended in the patients with residual head tilt, passive rotation deficit, or lateral bending over 15° at the age of 6 months [5].

The timing for surgery is controversial. Canale et al. reported that full recovery of facial asymmetry after the age of four is difficult to achieve [19]. Characteristically, there are flattening of the occiput contralaterally and depression of the malar prominence ipsilaterally, with downward displacement of the ear, eye and mouth on the affected side. Provided that the surgery is done in the immature patients, these skeletal deformities may improve following surgery [20].

Lee et al. [16], Minamitani et al. [21], and Chen et al. [12] reported that late release of sternocleidomastoid muscle for the patients of more than six years of age could yield acceptable results. All of these studies were carried out mostly on older children not adults. Although, we also agree with these authors and suggest that satisfactory results will be obtained regardless of the patient's age if a suitable operation and rehabilitation is performed and improving the skeletal deformity is not a significant concern.

In the series of 18 delayed cases of 6 to 22 (average 11) years of age, Chen and Ko found that preoperative open mouth radiograph of the odontoid process in 16 patients revealed asymmetry of articular facets of the axis and tilt of the odontoid process to the side of torticollis [12]. They observed complete improvement of the tilt of the odontoid process after surgery; where as asymmetry of articular facets of the axis persisted. In addition, two patients showed no improvement in fascial asymmetry. They used Lee's scoring system and with a mean follow-up of 5 years, they could achieve excellent result in 7, good in 3, fair in 6 and poor in 2 patients. It is recommended to perform bipolar release in patients with persistent deformities.

The only paper we could find in the literature concerning surgery in adult CMT belonged to Ippolito and Tudisco [22]. In a group of patients over the age of 26, they reported that, although there was no resolution in facial asymmetry, there was improvement in the neck movement of all patients, and that there were no complications. In contrast, Ling maintains that the benefit of treatment is limited over the age of 5 and that the complication rate is high [23].

Although, there are various surgical procedures for CMT, unipolar and bipolar release are the most popular. Bipolar releases are usually used in older ones with severe deformity. Wirth et al. in a review of 55 patients with an average follow-up of 15 years after surgical release recommended that biterminal release should be performed at the age of 3–5 years in all patients who do not respond to non-operative treatment [24]. Although, it was reported that bipolar release combined with Z-plasty can preserve the normal v-contour of sternocleidomastoid muscle in the neckline [25], we similar to Chen and Ko [12] observed no loss of normal contour of sternocleidomastoid muscle in the patients received a bipolar release without Z-plasty. With meticulous repair of the platisma, loss of cervical column does not occur clinically. Therefore, we confirmed that Z-plasty must not be considered to be necessary in neglected old ones. We also observed no patient with overcorrection in our series.

Based on our experience with adult cases, we believe that in the time of distal operation, both sternal and clavicular heads must be released, even though the clavicular head seems trivial. Due to muscular adhesion to the fascia, we not only release the muscular tissue, but also excise about 2 cm of it. After resecting both clavicular and sternal heads, the patient's head must be turned to the lateral side and all the firm touched fascial bands released.

As the cases we described were skeletally mature with long standing deformity, one does not expect significant regression of the cervico-thoracic scoliosis on follow-up radiographs. Obviously, to determine the influence of a bipolar release on the ultimate curve regression, longer follow-up is needed.

## Conclusion

In conclusion, patients with congenital muscular torticollis can benefit from surgical treatment even in adulthood. Surgical bipolar sectioning of the sternocleidomastoid muscle should be considered even in adults with irreversible facial and skeletal deformities. The surgery restores the range of neck motion and resolves the head tilt; therefore it can improve the quality of life. This procedure is an effective and relatively complication-free method.

## Competing interests

The authors declare that they have no competing interests.

## Authors' contributions

FOK participated in the sequence alignment and drafted the manuscript. EGH participated in the sequence alignment. RS participated in the design of the study. MM conceived of the study, and participated in its design and coordination. All authors read and approved the final manuscript.

## Consent

Written informed consent was obtained from the patient (case 3) for publication of the images. A copy of the written consent is available for review by the Editor-in-Chief of this journal.

## Pre-publication history

The pre-publication history for this paper can be accessed here:


